# Preoperative bevacizumab does not increase complications following cytoreductive surgery and hyperthermic intraperitoneal chemotherapy

**DOI:** 10.1371/journal.pone.0243252

**Published:** 2020-12-03

**Authors:** Bradley H. King, Joel M. Baumgartner, Kaitlyn J. Kelly, Rebecca A. Marmor, Andrew M. Lowy, Jula Veerapong

**Affiliations:** 1 School of Medicine, University of California, San Diego, La Jolla, California, United States of America; 2 Division of Surgical Oncology, Department of Surgery, University of California, San Diego, La Jolla, California, United States of America; Peter MacCallum Cancer Institute, AUSTRALIA

## Abstract

**Background:**

Preoperative bevacizumab has been reported to increase postoperative complication risk following cytoreductive surgery and hyperthermic intraperitoneal chemotherapy (CRS/HIPEC). We sought to review our experience with preoperative bevacizumab in patients undergoing CRS/HIPEC for peritoneal surface malignancy.

**Methods:**

This is a retrospective review of patients who received neoadjuvant systemic therapy with or without bevacizumab prior to CRS/HIPEC at a high-volume academic center from 2007–2018.

**Results:**

Of 499 patients, a total of 88 patients received neoadjuvant chemotherapy alone (n = 34) or in combination with bevacizumab (n = 54) within 3 months prior to CRS/HIPEC. No differences existed in 60-day major morbidity (17.6 vs. 16.7%, p = 0.81) or 60-day mortality (0 vs. 0%) between the two cohorts, and neoadjuvant bevacizumab was not associated with increased odds of overall complications (OR 0.86, 95% CI 0.35–2.09, p = 0.73) or major morbidity (OR 0.86, 95% CI 0.24–3.00, p = 0.81). Stratifying patients by primary tumor origin and post-operative complications did not reveal any significant differences between the two treatment groups. In addition, progression-free survival (PFS) and overall survival (OS) were similar in both cohorts.

**Conclusions:**

Preoperative bevacizumab is not associated with increased morbidity or mortality following CRS/HIPEC. Neoadjuvant therapy employing this biologic agent is safe and should not be a deterrent for aggressive cytoreduction with curative intent.

## Introduction

Cytoreductive surgery with hyperthermic intraperitoneal chemotherapy (CRS/HIPEC) is commonly employed in the management of peritoneal surface malignancies. Surgical debulking reduces the macroscopic tumor burden while regionally-administered chemotherapy targets residual microscopic peritoneal disease, yielding survival benefits over systemic chemotherapy and palliative surgery [1]. Depending on the histopathologic subtype, neoadjuvant systemic therapy is often given to patients for reduction of tumor volume and demonstration of biology to assess candidacy for surgery. Such upfront treatment may involve the use of biologic agents.

Bevacizumab (Avastin®), a monoclonal antibody directed against vascular endothelial growth factor (VEGF), is an agent commonly employed in stage IV colorectal cancer patients. Overall survival, progression-free survival, response rate, and duration of response are enhanced in metastatic colorectal cancer patients when bevacizumab is added to a regimen of fluorouracil, leucovorin, and irinotecan (FOLFIRI) [2]. Additionally, combining bevacizumab therapy with oxaliplatin marginally increases progression-free survival in patients with metastatic colorectal cancer and has utility in refractory metastatic colorectal cancer when used alongside fluorouracil, leucovorin, and oxaliplatin (FOLFOX) [3, 4]. Vascular endothelial growth factor mediates pathologic angiogenesis and is also implicated in crucial components of the peritoneal metastatic cascade including the epithelial-to-mesenchymal transition, degradation of the submesothelial matrix, and ascites formation [5–8]. Direct inhibition of this protein may slow such processes and enhance the preoperative tumor response in peritoneal disease [9].

Cytoreductive surgery often requires multiple anastomoses and carries with it feared complications including leaks, fistulae, perforation, and severe post-operative bleeding [10]. With a relatively long half-life in serum (~ 20 days), bevacizumab is commonly held 6–8 weeks prior to surgery to allow proper time for drug clearance. In patients undergoing surgery for metastatic colon cancer, bevacizumab has been shown to increase the incidence of spontaneous bowel perforation, arterial thromboembolic events, grade 3–4 bleeding, and postoperative wound-healing complications [11–14].

The data regarding the safety of neoadjuvant bevacizumab prior to CRS/HIPEC are mixed. A French multicenter study found that the addition of bevacizumab to neoadjuvant chemotherapy in stage IV colorectal cancer patients undergoing CRS/HIPEC resulted in a twofold increase in early morbidity, with a notable increase in enteric fistulae [15]. A retrospective study examining neoadjuvant bevacizumab with chemotherapy prior to CRS/HIPEC suggested improved outcomes with increased overall survival from 22 months to 39 months with the addition of bevacizumab to systemic therapy [16]. Notably, there was no difference in postoperative morbidity in this patient cohort.

The present study compares morbidity outcomes in CRS/HIPEC patients receiving neoadjuvant systemic chemotherapy alone or in combination with bevacizumab within three months of surgery in order to assess the safety of this biologic agent.

## Methods

### Patient selection

Upon institutional review board approval for this project by the University of California, San Diego Human Research Protections Program (Project #140754CX), a de-identified retrospective database was accessed from September 2015 to October 2020. Four hundred ninety-nine patients who underwent CRS/HIPEC between June 2008 and July 2018 at UC San Diego Health were selected for the study. Patient data were obtained from a review of physical and electronic medical records, which included demographic data and preoperative, operative, and postoperative variables. Patients who received neoadjuvant chemotherapy alone or in combination with bevacizumab within 3 months prior to surgery for peritoneal disease of colorectal cancer, appendiceal cancer, or diffuse malignant peritoneal mesothelioma origin were included in this study. Patients with alternative primary tumor origin or who experienced latency of more than 90 days from the date of last chemotherapy to surgery were excluded from analysis. Additionally, patients with incomplete chart data including omitted morbidity outcomes were not considered in this study. Chemotherapy regimens differed based on the best course of clinical management. Throughout the entire study period, institutional policy suggested that bevacizumab be held for 6–8 weeks prior to surgery. The primary outcome measure was overall morbidity after surgery. Grade III/IV morbidity,60-day mortality, progression-free survival (PFS), and overall survival (OS) were secondary outcomes.

### Operative management

All patients underwent CRS followed immediately by HIPEC per the standardized technique performed at our institution. The extent of peritoneal metastases was assessed at the time of surgery and recorded according to the peritoneal cancer index (PCI). The completeness of cytoreduction score (CC score) was used after cytoreduction to assess residual, unresected disease: CC-0 had no visible residual disease, CC-1 had residual tumor nodules up to 2.5 mm, CC-2 had residual nodules up to 2.5 cm, and CC-3 had residual nodules > 2.5 cm. Hyperthermic intraperitoneal chemotherapy was subsequently performed using a closed abdomen perfusion technique with 3–6 liters of warmed perfusate and intraperitoneal chemoperfusion for 90 minutes with a goal intraperitoneal hyperthermia of 42^⁰^C. Patients with appendiceal, colorectal, and small bowel primary tumors were given 10 mg/L perfusate of intraperitoneal mitomycin C. Patients with diffuse malignant mesothelioma were administered 50 mg/m^2^ cisplatin and 15 mg/m^2^ doxorubicin.

### Statistical analysis

Results of the statistical analysis are expressed as medians with first and third quartiles or proportions and percentages unless otherwise indicated. The two patient groups were probed for statistical differences using Student’s *t* test and the Fisher exact test, with a p-value of < 0.05 considered significant. Progression-free and overall survival were calculated from the time of CRS/HIPEC using the Kaplan-Meier method. A log-rank test was used to compare PFS/OS among groups. All statistical analyses were performed using SPSS Statistics Version 25.0 (IBM, Armonk, New York).

## Results

### Clinical and surgical data

The clinical and surgical characteristics of the 88 patients receiving either systemic chemotherapy alone (systemic chemotherapy group, n = 34) or in combination with bevacizumab (bevacizumab group, n = 54) are listed in Table 1. Mean albumin in the bevacizumab group (4.33 g/dL, SD 0.33) was significantly higher than in the systemic chemotherapy group (4.09 g/dL, SD 0.51), though each were in the normal range. A greater number of patients receiving bevacizumab had a primary colorectal tumor origin (36, 66.7%) than those given systemic chemotherapy alone (14, 41.2%) (p = 0.03). In contrast, fewer mesothelioma patients were contained in the bevacizumab group (2, 3.7%) than in the systemic chemotherapy group (15, 44.1%) (p < 0.001). Complete cytoreduction (CC-0 and CC-1) was attained in approximately equal proportion within the bevacizumab group (52, 96%) and the systemic chemotherapy group (29, 91%) (p > 0.05). Charlson comorbidity index (CCI) (0 vs. 0, p = 0.98) and median number of anastomoses (1 vs. 1, p = 0.65) did not significantly differ between the two groups. Of note, a greater number of weeks elapsed between the last chemotherapy dose and CRS/HIPEC in the bevacizumab group (9.2 [*Q*_*1*_ 8.1, *Q*_*3*_ 10.5]) than in the systemic chemotherapy group (7.2 [*Q*_*1*_ 5.4, *Q*_*3*_ 9.0]) (p = 0.001). The shortest interval from bevacizumab withdrawal to surgery was 35 days, while the shortest interval from chemotherapy cessation was 21 days.

**Table 1:** Characteristics of patients receiving systemic chemotherapy alone (‘systemic chemotherapy’) or in combination with bevacizumab (‘bevacizumab’) prior to CRS/HIPEC.

Characteristic	Systemic chemotherapy (n = 34)*	Bevacizumab	p
		(n = 54)*	
Age (years)	53.2 (45.5, 58.8)	51.8 (46.6, 63.2)	0.31
BMI	28.0 (22.0, 31.4)	25.5 (22.6, 28.5)	0.13
Male gender	18 (52.9%)	23 (42.6%)	0.39
Mean albumin (st. dev.)	4.09 (0.51)	4.33 (0.33)	0.02
Charlson comorbidity index	0 (0, 0)	0 (0, 0)	0.98
PC synchronous	24 (70.6%)	33 (61.1%)	0.49
Peritoneal cancer index	12.5 (7.5, 17.8)	11 (6.3, 15.0)	0.24
Primary tumor origin			
Colorectal	14 (41.2%)	36 (66.7%)	0.03
Appendiceal	5 (14.7%)	16 (29.6%)	0.13
Mesothelioma	15 (44.1%)	2 (3.7%)	~ 0
Completeness of cytoreduction			
CC-0	20 (58.8%)	43 (79.6%)	0.05
CC-1	9 (26.5%)	9 (16.7%)	0.29
CC-2	5 (14.7%)	2 (3.7%)	0.10
Number of anastomoses	1 (0, 2)	1 (0, 2)	0.65
Weeks from last CTX dose to surgery	7.2 (5.4, 9.0)	9.2 (8.1, 10.5)	0.001
Duration of surgery (min)	457 (354, 536)	425 (323, 518)	0.36
Estimated blood loss (mL)	150 (81, 375)	125 (100, 288)	0.65

* Median (quartiles) values are shown unless otherwise indicated.

### Post-operative complications

The worst 60-day Clavien-Dindo morbidity experienced by patients, as well as 60-day mortality, are shown in Table 2. No patients died within 60 days of CRS/HIPEC in either treatment group. The overall morbidity rate was 62.5%, with no significant difference between the bevacizumab and systemic chemotherapy groups (OR = 0.86, 95% CI 0.35–2.09, p = 0.73). Similarly, Grade III/IV morbidity was comparable (OR = 0.81, 95% CI 0.24–3.00, p = 0.81). The two groups had similar incidence of complications including anastomotic/bowel leak or fistula, ileus/delayed gastric emptying (DGE), intra-abdominal abscess formation, wound infection, DVT/PE, postoperative transfusion, and return to OR (Table 3). Of note, only patients who received one or more anastomoses were considered in the calculation of leak/fistula rate.

**Table 2:** Morbidity and 60-day mortality in patients receiving systemic chemotherapy alone or in combination with bevacizumab.

	Systemic chemotherapy (n = 34)	Bevacizumab (n = 54)	Odds ratio*	p
Overall morbidity	22 (64.7%)	33 (61.1%)	OR = 0.86 95% CI: 0.35–2.09	0.73
Grade III/IV morbidity	6 (17.6%)	9 (16.7%)	OR = 0.86 95% CI: 0.24–3.00	0.81
60-day mortality	0 (0%)	0 (0%)	--	--

* Odds ratios and associated p-values were calculated by comparing patients who experienced complications to those without any morbidity in the bevacizumab vs. systemic chemotherapy groups.

**Table 3:** Postoperative complications following CRS/HIPEC.

Complication	Systemic chemotherapy (n = 34)	Bevacizumab (n = 54)	p
Leak/fistula	2 (8.3%)	2 (5.7%)	0.70
Ileus/delayed gastric emptying (DGE)	4 (11.8%)	10 (18.5%)	0.55
Intra-abdominal abscess	3 (8.8%)	5 (9.3%)	~ 1
Wound infection	2 (5.9%)	4 (7.4%)	~ 1
DVT/PE	0 (0%)	2 (3.7%)	0.52
Postoperative transfusion	6 (17.6%)	12 (22.2%)	0.79
Return to OR	2 (5.9%)	3 (5.6%)	~ 1

**Fig 1:**
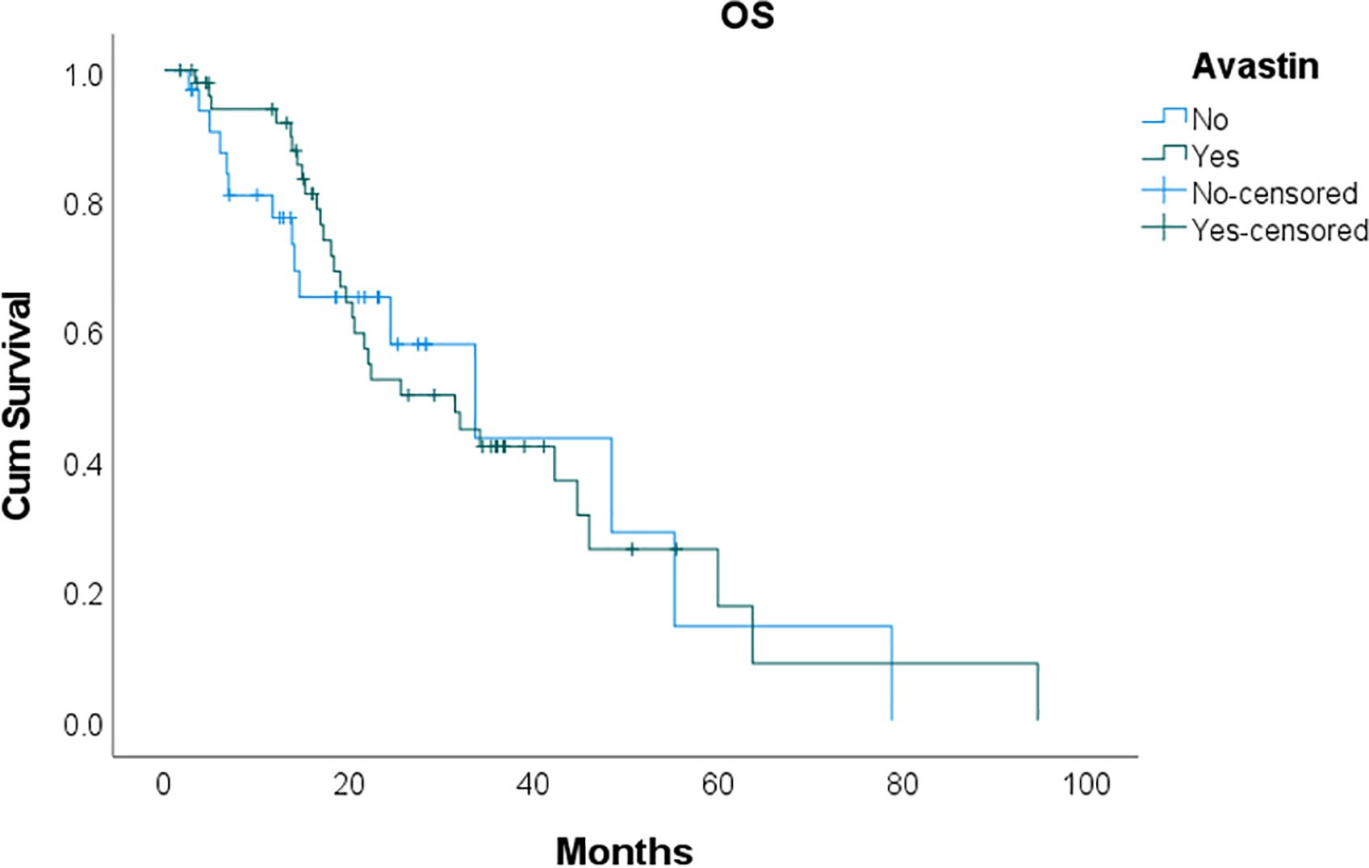
Kaplan-Meier curves of overall survival (OS) and progression-free survival (PFS). No significant difference in OS or PFS was observed between the bevacizumab and systemic chemotherapy groups.

**Fig 2:**
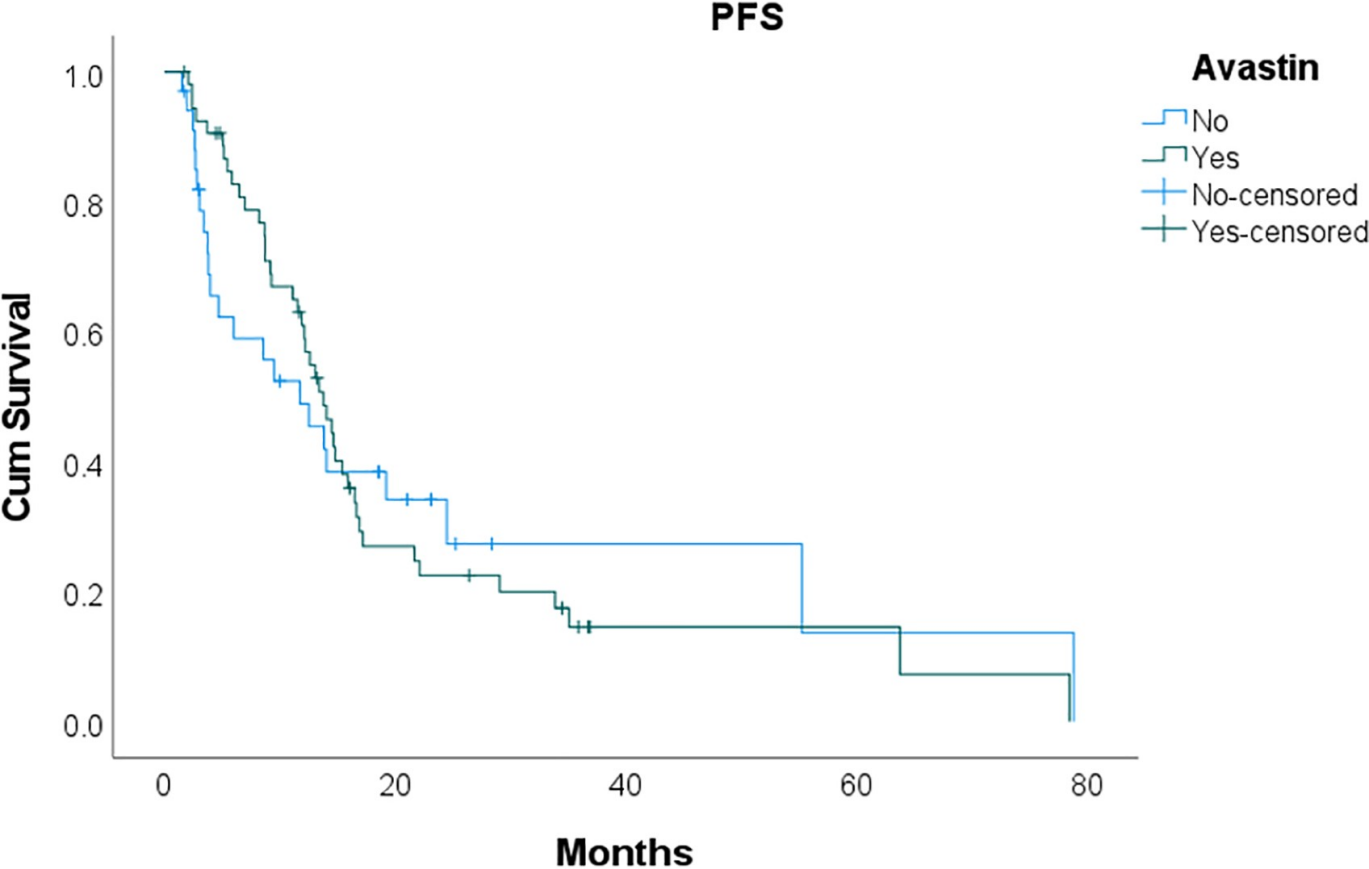
Kaplan-Meier curves of overall survival (OS) and progression-free survival (PFS). No significant difference in OS or PFS was observed between the bevacizumab and systemic chemotherapy groups.

Kaplan-Meier curves constructed for OS and PFS of the two patient cohorts are shown in Figs 1 and 2. Overall survival in the bevacizumab group (median OS 31.5 months, 95% CI 19.0–44.1 months) did not differ significantly from that of the systemic chemotherapy group (median OS 33.7 months, 95% CI 15.2–52.2 months; p = 0.85). Moreover, PFS was similar between the bevacizumab (median PFS 13.8 months, 95% CI 11.2–16.4 months) and systemic chemotherapy groups (median PFS 11.8 months, 95% CI 5.0–18.5 months; p = 0.96).

## Discussion

Despite continued advances in systemic therapy regimens, patients with peritoneal surface malignancies derive the most meaningful survival benefit from complete cytoreductive surgery (CC-0/CC-1). The patient’s fitness for surgery is often based on several factors, including performance status, disease burden, and tumor biology. For more aggressive histopathologic cancer subtypes or grades, a neoadjuvant approach with chemotherapy remains a mainstay to select patients for a surgical procedure with inherent morbidity. Favorable, or at least stable, response to neoadjuvant therapy is required to move on to CRS/HIPEC. Bevacizumab is frequently employed as an adjunct in the neoadjuvant approach, but conflicting evidence has been reported regarding the safety of its administration before cytoreductive surgery and HIPEC. In the French retrospective analysis of 182 patients, patients who received neoadjuvant bevacizumab experienced 2-fold greater 30-day major morbidity (Clavien-Dindo grade III/IV) compared to those treated with systemic chemotherapy alone, though mortality remained unaffected [15]. However, no difference in major morbidity was observed in study examining a cohort of 26 patients receiving neoadjuvant chemotherapy with or without bevacizumab for carcinomatosis from high-grade appendiceal cancer [17]. Another study of 61 patients who underwent CRS and HIPEC found that neoadjuvant bevacizumab was not associated with an increase in major morbidity compared to chemotherapy alone and conferred a nearly doubled overall survival benefit [16]. The findings of this present study provide support for the safety of neoadjuvant bevacizumab, with comparable rates in 60-day overall and major morbidity to conventional chemotherapy.

The bevacizumab and systemic chemotherapy groups differed in several pre-operative metrics. Mean albumin was significantly lower in the systemic chemotherapy group (4.09 vs. 4.33, p = 0.02). However, this is not likely of clinical significance as neither group was hypoalbuminemic [18]. Though the distribution of primary tumor origin was variable between the two patient groups, stratifying by primary malignancy revealed no differences in morbidity or mortality. Importantly, the rate of specific post-operative complications, including those related to major bleeding events, were similar in each patient group. Patients in the bevacizumab group had a longer interval before undergoing CRS/HIPEC, likely reflecting our institutional bias of waiting up to 8 weeks after the administration of the last dose of bevacizumab prior to a major abdominal procedure.

One of the more feared complications of bevacizumab includes anastomotic breakdown with resultant enteric leak or enterocutaneous fistula. Our study demonstrates a low rate leak or fistula rate in both groups, with the bevacizumab group at 5.7% and systemic chemotherapy group at 8.3%. Moreover, there were no differences in outcomes between the two groups. Our data show that, even after stratifying by primary malignancy, no significant differences existed in 60-day overall complication rate, 60-day major morbidity, or 60-day mortality between patients receiving bevacizumab or systemic chemotherapy alone. Median OS and PFS were also similar in both groups, further suggesting the overall safety of bevacizumab therapy prior to CRS-HIPEC, particularly since this agent is typically administered to patients with higher baseline disease burden. Prospective data from ongoing clinical trials including “BEV-IP: Perioperative Chemotherapy with Bevacizumab for Colorectal Carcinomatosis” (NCT02399410) will be critical in validating these results and guiding patient management.

The limitations of this study include a relatively small sample size and the retrospective nature of the data collection. There was also an imbalance in makeup of the primary tumor origins in each group, with the bevacizumab group being composed of more colorectal patients and fewer diffuse malignant peritoneal mesotheliomas proportionally.

## Conclusion

Bevacizumab remains a routine therapeutic agent in the management of metastatic colorectal cancer and mesothelioma given studies demonstrating survival benefit. This study corroborates the safety of utilizing bevacizumab in the neoadjuvant setting prior to CRS/HIPEC provided that there is an appropriate interval between cessation of therapy and commencement of surgery.
